# Bethesda Endoscopic Retrograde Cholangiopancreatography Skill Assessment Tool (BESAT)‐Based Simulator Training Using a Novel Dry Endoscopic Retrograde Cholangiopancreatography Model Improves Technical Proficiency and Self‐Efficacy: A Prospective Study

**DOI:** 10.1002/deo2.70382

**Published:** 2026-09-01

**Authors:** Yutaka Hatayama, Takeshi Kanno, Tetsuya Takikawa, Ryotaro Matsumoto, Yutaro Arata, Suguo Suzuki, Kaname Uno, Kazuhiro Kikuta, Tomoyuki Koike, Atsushi Masamune

**Affiliations:** ^1^ Division of Gastroenterology Tohoku University Graduate School of Medicine Miyagi Japan; ^2^ R & D Division of Career Education for Medical Professionals Medical Education Center Jichi Medical University Tochigi Japan; ^3^ Graduate Medical Education Center Tohoku University Hospital Miyagi Japan; ^4^ Division of Promotion for Gastroenterological Medical Innovation Tohoku University Miyagi Japan

**Keywords:** education, endoscopic retrograde cholangiopancreatography, endoscopic sphincterotomy, simulator, training

## Abstract

**Objectives:**

Endoscopic sphincterotomy (EST) is a high‐risk procedure with a steep learning curve. We previously developed a novel dry simulator capable of reproducing bleeding after an inappropriate incision. This study aimed to evaluate the effectiveness of a structured educational program using the simulator within the Bethesda Endoscopic retrograde cholangiopancreatography (ERCP) Skill Assessment Tool (BESAT) framework.

**Methods:**

This prospective study implemented a standardized training sequence: (1) preliminary video‐based learning; (2) an initial attempt at cannulation, EST, and biliary stone extraction; (3) BESAT‐based expert feedback focusing on six key technical tips; (4) a second attempt. The primary outcome was the change in EST success, which is defined by an incision in the 11–12 o'clock direction with appropriate length and no bleeding, before and after feedback. Secondary outcomes were procedure completion, including stone removal within 15 min, and subjective self‐assessments of procedural understanding and confidence.

**Results:**

Thirty gastroenterology trainees were enrolled. The EST success rate increased from 43.3% to 76.7% (*p* < 0.05). The completion rate of the procedure tended to increase from 46.7% to 70.0% (*p* = 0.07). Subjective self‐confidence improved from a median of 24.0–53.0 after training and remained at 53.5 at 3 months; other self‐assessed domains also improved and were sustained. Post‐hoc analysis showed that initial EST success was associated with >50 prior ERCP cases (odds ratio = 7.86, 95% confidence interval: 1.31–47.0, *p* < 0.05).

**Conclusions:**

A structured simulator‐based program enables trainees to experience failure and improves simulator‐based EST performance with sustained gains in self‐efficacy.

**Trial Registration**: The trial was prospectively registered in the University Hospital Medical Information Network Clinical Trials Registry (UMIN000044823).

## 1 Introduction

Endoscopic retrograde cholangiopancreatography (ERCP) and endoscopic sphincterotomy (EST) are essential yet technically challenging procedures. Acute cholangitis, the primary indication for these procedures, is a life‐threatening condition with reported mortality rates ranging from 2.7% to 24%, even in the modern era [1, 2]. Mastering these procedures requires precise manipulation of the side‐viewing duodenoscope and coordination of various therapeutic devices. The anatomical complexity within the duodenum, combined with the inverted field of view due to side‐viewing optics, creates a steep learning curve that is difficult to navigate during the initial training.

EST is essential for stone extraction and biliary drainage, but is associated with a high risk of adverse events among all ERCP‐related procedures [3]. In Japan, the reported incidence of EST complications is 1.34%, with post‐ERCP pancreatitis, hemorrhage, and perforation being the primary concerns. For procedural safety, Japanese guidelines recommend an incision between the 11 and 12 o'clock positions, reaching the hooding fold without exceeding the oral protrusion [4]. Anatomical studies have shown that the 11 o'clock sector usually has fewer arterial vessels near the major papilla, minimizing the risk of severe hemorrhage [5]. Despite these benchmarks, achieving directional accuracy and an appropriate incision length remains challenging for novice endoscopists.

Currently, ERCP/EST training predominantly relies on on‐the‐job training (OJT) with actual patients. The American Society for Gastrointestinal Endoscopy recommends approximately 200 procedures, including at least 40 ESTs, before independent practice [6]. However, OJT inherently poses risks to patient safety and often occurs in high‐pressure scenarios that preclude the calm, standardized environment necessary for structured feedback [7, 8, 9]. In addition, anatomical variation between patients makes a uniform learning experience difficult. To address these challenges, several simulator models have been developed [10, 11, 12, 13]. Biologic “wet” models provide high fidelity but involve significant ethical concerns and preparation complexity. Conventional “dry” models are more accessible, yet they often lack the realism necessary to simulate critical complications, such as bleeding [14]. Based on our previous development and educational validation of endoscopic hemostasis simulators [15, 16], we developed a novel dry ERCP/EST model featuring a luminal tract designed to replicate realistic endoscope positioning and operator posture. This model incorporates a hydrogel‐based papilla with artificial vessels to reproduce bleeding when an incorrect incision is made, and its high realism has been validated in preliminary evaluations [17].

The Bethesda ERCP Skill Assessment Tool (BESAT) is a rubric‐based framework that defines the technical components required for safe procedural execution. Its validity in distinguishing expert and novice endoscopists provides clear milestones in technical proficiency [18]. We developed a simulation‐based training (SBT) program using our dry ERCP simulator with learning objectives derived from the BESAT framework. This prospective study aimed to evaluate the efficacy of this program and its impact on the technical proficiency and self‐efficacy of non‐expert trainees.

## 2 Methods

### 2.1 Study Design and Participants

This prospective educational study was conducted between May 2022 and March 2023. The eligible participants were non‐expert endoscopists (Gastrointestinal trainees) at Tohoku University Hospital who did not have board certifications from the Japan Gastroenterological Endoscopy Society. Conversely, experts, defined as board‐certified trainers or individuals with ≥10 years of experience as an endoscopist, were excluded. To specifically target learners in the early to intermediate technical acquisition phases, we also excluded trainees with prior clinical experience of ≥100 ERCP procedures. Additional exclusion criteria were withdrawal of consent and safety concerns. The study protocol was approved by the Ethics Committee of the Tohoku Medical Megabank Organization, Tohoku University, on March 15, 2021 (approval number 2020‐4‐195) and conducted in accordance with the Declaration of Helsinki. The trial was prospectively registered in the University Hospital Medical Information Network (UMIN) Clinical Trials Registry (UMIN000044823).

### 2.2 Simulator and Therapeutic Devices

**FIGURE 1:**
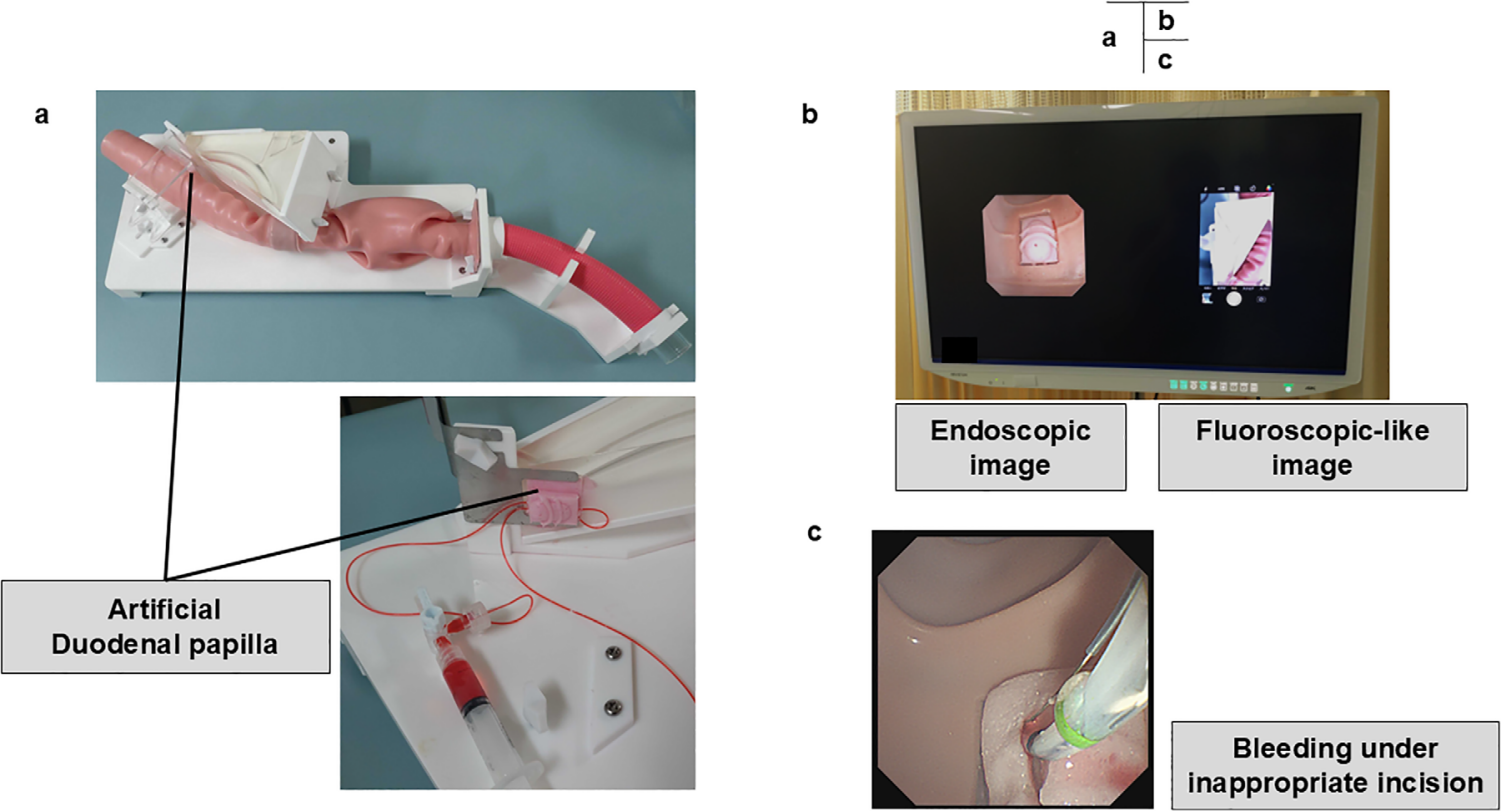
The simulator (Medical Rising STAR, ERCP/EST type). (a) Overview of the novel complication‐reproducible ERCP/EST simulator. The model consists of a reusable upper gastrointestinal tract constructed from polyethylene foam and silicone and a disposable hydrogel‐based papillary section with 0.8‐mm artificial vessels. It is designed to reproduce realistic endoscope positioning and operator posture for achieving a frontal view of the papilla in the prone position. (b) Representative view showing simultaneous visualization of the endoscopic image and fluoroscopic‐like image projected from a mobile device on the submonitor during device manipulation. (c) Representative view of pulsatile bleeding after an inappropriate EST incision. The artificial vessels are designed to bleed when the incision deviates from the recommended 11–12 o'clock sector or exceeds the oral protrusion. ERCP, endoscopic retrograde cholangiopancreatography; EST, endoscopic sphincterotomy.

The simulator (Medical Rising STAR, ERCP/EST type) was developed through an industry–academia partnership. It consists of a reusable upper gastrointestinal tract and a disposable papillary section designed to reproduce realistic ERCP/EST maneuvers and bleeding after inappropriate incision [17] (Figure 1 and Video S1).

Standard endoscopic devices were used in all sessions: a duodenoscope (JF‐260 V; Olympus, Tokyo), 0.025‐inch guidewire (VisiGlide 2; Olympus), cannula (StarTip V; Olympus), sphincterotome (CleverCut 3 V; Olympus), and basket catheter (Flower Basket V; Olympus). For EST, an electrosurgical unit (VIO‐3; ERBE, Tübingen, Germany) was used in ENDO CUT mode (effect 2).

### 2.3 Structured Training Protocol

The program followed a standardized four‐step sequence:

Standardized preliminary instruction: Trainees watched a standardized instructional video explaining the procedural flow of ERCP/EST, side‐viewing endoscope manipulation, and the mechanics of the used devices.

Initial attempt: Under a scenario of acute cholangitis with common bile duct stones, the participants attempted the full procedural sequence, including biliary cannulation, guidewire placement, EST, and stone extraction, without instructor guidance.

Educational intervention: Expert endoscopists provided a 10‐min mini‐lecture and individualized feedback based on the BESAT framework [18], focusing on predefined technical points for safe and effective ERCP/EST (Supporting Information 1). Second attempt: Trainees repeated the entire procedural sequence immediately after the intervention.

### 2.4 Outcome Measures

The primary objective outcome was EST success rate. Success was defined based on the Japanese guidelines for EST [4] and operationalized in the simulator as achieving a medium‐length incision in the 11–12 o'clock direction, reaching the hooding fold without exceeding the oral protrusion and without causing artificial vessel bleeding (Figure S1). Since the papillary portion of the simulator was standardized, incision adequacy was evaluated based on predefined anatomical landmarks rather than millimeter‐based measurements. These criteria were explained before the initial attempt using study materials and the instructional video. Secondary objective metrics included the successful stone extraction rate within 15 min and total procedure time from the initial frontal view of the papilla to final stone removal. Two board‐certified trainers served as evaluators. They performed the technical assessments in real time during the simulator training and knew whether it was the first or second attempt. In the event of a disagreement, the final judgment would be reached through discussion between the two evaluators.

Subjective proficiency was assessed using a Visual Analog Scale (VAS, 0–100) across two domains: (i) self‐confidence regarding duodenoscope manipulation and independent procedural success (Q1–Q3), and (ii) understanding of therapeutic devices, such as the sphincterotome and basket catheter (Q4‐Q5). These assessments were repeated 3 months after training to evaluate long‐term retention.

Post‐hoc analysis was performed using logistic regression to identify the relationship between trainee background and initial EST success. This analysis evaluated the clinical relevance of the model by determining whether prior real‐world experience predicted performance on the simulator.

### 2.5 Sample Size Calculations and Statistical Analyses

This study was designed as a pilot prospective educational study, and the sample size was pragmatically determined with reference to prior simulation‐based research [16], because an expected effect size for the objective binary outcome of EST success on the simulator was difficult to prespecify. As a practical reference, G*Power 3.1 showed that 19 participants would be required for a Wilcoxon signed‐rank test assuming an effect size of 0.7, an alpha error of 0.05, and a power of 0.8. We therefore set the target sample size at 30 participants. The subjective scores were compared using the Wilcoxon signed‐rank test. Friedman's test was used for comparisons across three time points, followed by the Wilcoxon signed‐rank test with Bonferroni correction. Objective success rates were analyzed using McNemar's test. Continuous variables in the post‐hoc analysis comparing two independent groups were analyzed using the Mann‐Whitney U test, and categorical variables were compared using Fisher's exact test. Statistical significance was set at *p* < 0.05. All statistical analyses were performed using EZR version 1.53 [19].

## 3 Results

### 3.1 Participant Characteristics

Thirty gastroenterology trainees (24 men and six women) were enrolled between May 2022 and March 2023 after providing oral informed consent. No dropouts or withdrawals occurred. The median age was 32 years, with a median time since graduation of 7 years. The median endoscopy training duration was 57.5 months (interquartile range [IQR], 44–102 months). While 94% of the participants had performed >50 endoscopic mucosal resections as the operator, their experience in advanced biliary procedures was limited: only 60% had performed >50 ERCPs, and 30% had performed >50 ESTs (Table 1).

**TABLE 1:** Background characteristics.

	Gastroenterology residents (*n* = 30)
Background characteristics	
Age, years, median (IQR)	32.0 (30.3–34.0)
Sex (Male/Female)	24/6
Postgraduate year, median (IQR)	7.0 (6.0–8.0)
Endoscopy training duration, months, median (IQR)	57.5 (44.0–102.0)
Experience of endoscopic procedures as an operator (cases)	0–10/11–50/51–100/100<
Endoscopic hemostasis	0/11/12/7
EMR	0/2/11/17
ESD	7/19/4/0
ERCP	3/9/13/5
EST	7/14/6/3

Abbreviations: EMR, endoscopic mucosal resection; ERCP, endoscopic retrograde cholangiopancreatography; ESD, endoscopic submucosal dissection; EST, endoscopic sphincterotomy; IQR, interquartile range.

### 3.2 Objective Technical Performance

**FIGURE 2:**
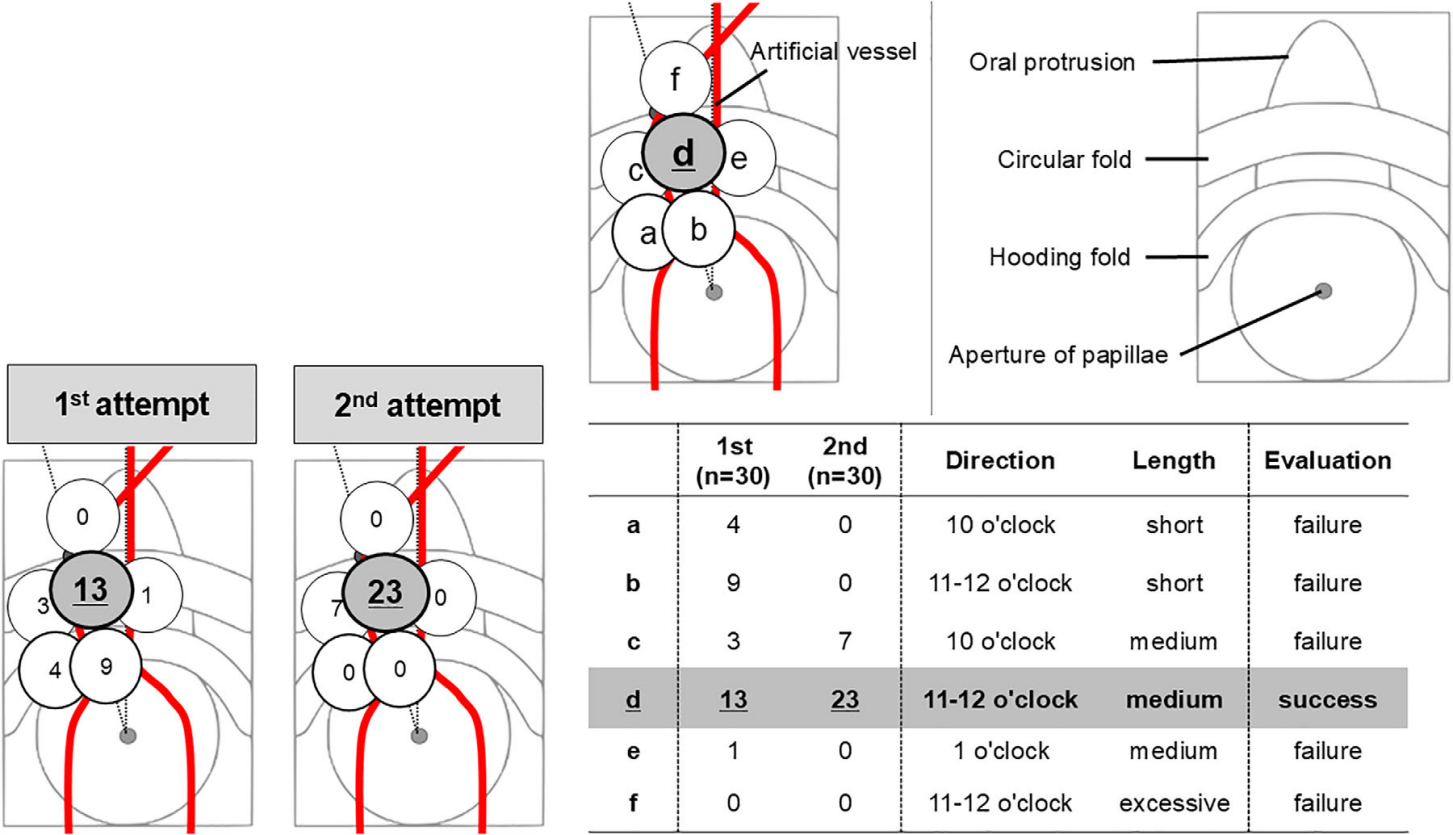
Comparative success rates of EST of the initial attempt (pre‐feedback) and the post‐feedback attempt. EST, endoscopic sphincterotomy.

The trainees’ technical performance significantly improved following the structured educational intervention. The success rate of EST increased from 43.3% (13/30) on the initial attempt to 76.7% (23/30) on the second attempt (Table 2; *p* < 0.05). Detailed analysis of the technical errors revealed that in the initial attempt, failures were predominantly due to “inadequate incision length” (*n* = 13) and “misdirection” (*n* = 8). Following expert feedback and the mini lecture, all 30 participants achieved the target incision length; however, misdirection (primarily toward the 10 o'clock position) persisted as the sole cause of failure in seven cases during the second attempt (Figure 2). The completion rate for the entire stone extraction procedure showed an upward trend from 46.7% to 70.0%, although it did not reach statistical significance (*p* = 0.07). For the 12 participants successful in both attempts, the median procedure time decreased significantly from 711.0 s (IQR 666.8–815.8) in the initial attempt to 583.0 s (IQR 487.3–744.3) in the second attempt (*p* < 0.05) (Table 2).

**TABLE 2:** Objective technical performance.

	Initial attempt (*n* = 30)	2nd attempt (*n* = 30)	*p*‐value
EST			
Success/Failure (success rate)	13/17(43.3%)	23/7 (76.7%)	<0.05^†^
Stone removal			
Success/Failure (success rate)	14/16 (46.7%)	21/9 (70.0%)	n.s. (0.07)^†^
			
Trainees with successful stone removal in both attempts	Initial attempt (*n* = 12)	2nd attempt (*n* = 12)	
Procedure time, seconds, median (IQR)	711.0 (666.8–815.8)	583.0 (487.3–744.3)	<0.05^*^

Abbreviations: EST, endoscopic sphincterotomy; IQR, interquartile range.

^†^ McNemar test.

* Wilcoxon signed‐rank test.

### 3.3 Subjective Impact and Long‐term Retention

**FIGURE 3:**
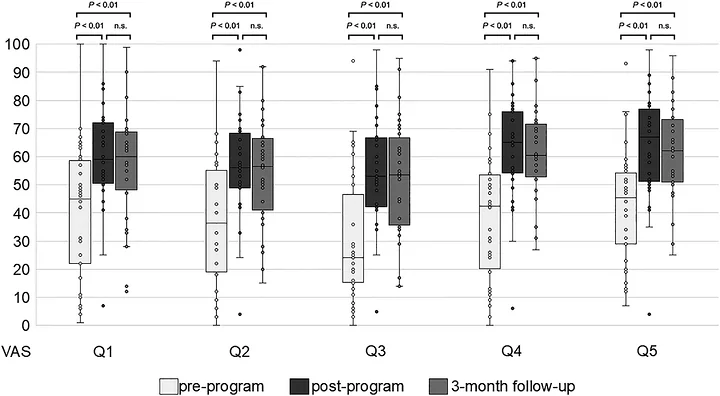
Retention of self‐confidence and device understanding at three points (Baseline, Post‐training, and 3‐month follow‐up) assessed using Visual Analog Scale (VAS).

The subjective VAS scores improved significantly across all assessed domains immediately after training (*p* < 0.01). Self‐confidence in performing independent EST and stone removal increased from a median of 24.0 at baseline to 53.0. Understanding the use of the sphincterotome device for the procedure improved from a median of 42.5–65.0. Regarding these psychological gains, at the 3‐month follow‐up, self‐confidence remained at 53.5 and understanding at 60.5 (*p* < 0.01 compared to baseline) (Figure 3).

### 3.4 Post‐hoc Analysis

EST success in the initial attempts was significantly associated with prior clinical experience of >50 ERCPs as the operator (odds ratio [OR] = 7.86, 95% confidence interval [CI]: 1.31–47.0, *p* < 0.05). Furthermore, initial success was also associated with baseline self‐confidence in duodenoscopic manipulation (Q1, OR = 1.04, *p* < 0.05) and biliary cannulation (Q2, OR = 1.04, *p* < 0.05). Younger age was marginally associated with initial EST success (Table 3).

**TABLE 3:** Factors associated with successful endoscopic sphincterotomy in the initial simulator attempt.

	Initial EST attempt		*p*‐Value	Univariate OR (95%CI)	*p*‐Value
	Success (*n* = 13)	Failure (*n* = 17)			
Background characteristics					
Age, years, median (IQR)	31.0 (30.0–32.0)	33.0 (31.0–34.0)	<0.05^*^	0.67 (0.45–1.00)	<0.05
Sex (Male/Female)	10/3	14/3	n.s.^†^	1.40 (0.23–8.42)	n.s.
Postgraduate year, median (IQR)	6.0 (6.0–8.0)	7.0 (6.0–8.0)	n.s.^*^	1.09 (0.64–1.87)	n.s.
Training duration, months, median (IQR)	48.0 (44.0–69.0)	60.0 (46.0–69.0)	n.s.^*^	1.00 (0.97–1.04)	n.s.
Background experience as an operator					
Endoscopic hemostasis (cases, 0–50/50<)	5/8	6/11	n.s.^†^	0.87 (0.20–3.90)	n.s.
EMR (cases, 0–50/50<)	1/12	1/16	n.s.^†^	0.75 (0.04–13.20)	n.s.
ESD (cases, 0–50/50<)	13/2	15/2	n.s.^†^	1.36 (0.17–11.20)	n.s.
ERCP (cases, 0–50/50<)	2/11	10/7	<0.05^†^	7.86 (1.31–47.00)	<0.05
EST (cases, 0–50/50<)	8/5	13/4	n.s.^†^	2.03 (0.42–9.89)	n.s.
Baseline self‐confidence (VAS), median (IQR)					
Q1. Duodenoscope manipulation	50.0 (47.0–60.0)	25.0 (17.0–46.0)	<0.05^*^	1.04 (1.00–1.08)	<0.05
Q2. Independent bile duct cannulation	49.0 (43.0–57.0)	27.0 (9.0–33.0)	<0.05^*^	1.04 (1.00–1.09)	<0.05
Q3. Independent EST and stone removal	29.0 (25.0‐53.0)	20.0 (13.0–24.0)	<0.05^*^	1.03 (1.00–1.07)	n.s. (0.08)
Q4. Use of the sphincterotome device	50.0 (30.0–58.0)	31.0 (11.0–47.0)	n.s. (0.08)^*^	1.03 (1.00–1.07)	n.s. (0.08)

Abbreviations: CI, confidence interval; EMR, endoscopic mucosal resection; ERCP, endoscopic retrograde cholangiopancreatography; ESD, endoscopic submucosal dissection; EST, endoscopic sphincterotomy; IQR, interquartile range; OR, odds ratio; VAS, visual analog scale.

* Mann‐Whitney U test, ^†^Fisher's Exact Probability Test.

## 4 Discussion

This prospective educational study demonstrated that a structured training program incorporating the BESAT rubric and a novel bleeding‐reproducible dry ERCP simulator significantly improved simulator‐based EST success and subjective confidence among trainees. An additional achievement of the study was the sustained retention of the psychological improvements over the 3‐month period, demonstrating that a single structured session can have a lasting impact on a trainee's perceived procedural readiness and self‐efficacy. Our program achieved a 33.4% increase in EST success, exceeding the benchmark proposed for valuable simulator‐based interventions [20].

A key strength of this program is that trainees can perform the entire procedural sequence, from scope positioning to stone retrieval, using actual devices in a simulator environment that permits safe failure. In traditional OJT, an expert instructor may take over the procedure as soon as a minor error occurs to maintain patient safety. This early takeover may limit trainees’ opportunities to experience technical errors and their consequences, which are important for learning. By reproducing pulsatile bleeding after an inappropriate incision, our simulator provides immediate and realistic feedback. This forces trainees to internalize the critical importance of the incision direction (11–12 o'clock) and length, which purely cognitive studies cannot replicate.

Our detailed error analysis proved particularly enlightening: while the educational intervention successfully corrected incision length errors for all participants, directional accuracy remained a challenge, even after feedback. This indicates that maintaining duodenoscopic stability and precise torque during high‐frequency current applications is a higher‐order skill that requires repetitive deliberate practice. The simulator uniquely facilitates this by allowing trainees to reset and attempt difficult maneuvers multiple times. Repeated learning opportunities using this simulator, together with longitudinal monitoring using objective assessment metrics, may help clarify whether these residual technical difficulties can be further improved.

The results showed a significant increase in EST success rate from 43.3% to 76.7%. In contrast, the completion rate for the entire stone extraction procedure showed an upward trend from 46.7% to 70.0%, but did not reach statistical significance (p = 0.07). This discrepancy likely stems from our study design, which defined success based on a strict 15‐min time limit for the entire sequence. Trainees who struggled with the initial EST phase often had insufficient time to retrieve the stones. However, for those who completed both attempts, the median procedure time decreased significantly. This indicates that the intervention primarily enhanced the efficiency of the EST phase, thereby streamlining the overall process.

The retention of subjective confidence and device understanding at 3 months was a pedagogical achievement. In real‐world clinical practice, learning about ERCP is often fragmented, stressful, and dictated by unpredictable patient volumes. By contrast, a structured program combining prior video‐based studies, hands‐on attempts, and targeted expert feedback based on the BESAT framework may help consolidate procedural understanding and self‐efficacy over time. However, these subjective outcomes should be interpreted cautiously, as they reflect self‐efficacy and perceived readiness rather than direct evidence of technical proficiency.

A perennial challenge in simulation‐based medical education is determining whether performance on a model reflects actual clinical skill [8, 9, 21]. Our post‐hoc analysis provided supportive evidence for the model's validity. In the present study, the initial EST attempt served as the baseline assessment before the BESAT‐based feedback intervention and was not intended to represent the educational effect of the simulator‐based training itself. The observed association between initial simulator success and prior clinical experience (>50 ERCP cases) suggests that performance on the model is linked to real‐world procedural experience. This finding supports the validity of the simulator as a training model with an appropriate level of technical difficulty. Because the model appeared to distinguish trainees with greater ERCP experience from less experienced trainees, it may serve as a proxy for aspects of clinical proficiency [22, 23]. Younger age was also marginally associated with initial success, perhaps reflecting more intuitive adaptation to the simulator interface, although this observation warrants further validation in larger cohorts. More broadly, further multifaceted evaluation is warranted to better establish the similarity of this simulator model to clinical EST.

Mastering safe maneuvers and risk management through simulation before attempting procedures on patients is fundamental to the “Patient Safety First” principle [24]. Our dry model adheres to the 3R principles (Replacement, Reduction, and Refinement) by providing a sustainable, hygienic, and reusable alternative to biological animal models [25, 26, 27]. Economically, although the initial investment in high‐fidelity simulators is a factor, the long‐term potential to reduce real‐world complications makes a compelling case for integrating such programs into standardized gastroenterology curricula.

### 4.1 Limitations

This study has several limitations. First, this was a single‐center study with a sample size of 30 trainees, which may limit the generalizability of the findings to other healthcare systems. In addition, as a pilot educational study, the sample size was set pragmatically with reference to prior simulation studies rather than being formally powered for the binary objective endpoint of EST success. Second, clinical skill improvement in real patients after the training session was not directly evaluated. At the same time, the association between prior clinical experience and initial simulator success suggests that the model retains at least a minimum degree of clinical reproducibility. Further investigation is required to determine how this simulator‐based program should complement traditional, real‐world, supervised EST training as an OJT. Third, although the model successfully replicates bleeding complications, it does not currently allow for hemostatic procedures, such as clipping or balloon compression, which are planned targets for next‐generation development [15, 16, 17]. Moreover, the current benchtop simulator does not fully reproduce several factors encountered in real ERCP/EST, including variable anatomy, respiratory movement, and unstable scope position. Fourth, the evaluation was performed in real time by trainers who also served as instructors and were not blinded to the timing of the attempts. This may have introduced evaluator bias in the objective success rates. Furthermore, because the second attempt was conducted immediately after the feedback session, short‐term familiarization or repetition cannot be ruled out. Fifth, subjective outcomes may have been influenced by psychological bias related to research participation, including the Hawthorne effect [28], rather than direct evidence of technical effectiveness. Sixth, objective technical performance was not reassessed at the 3‐month follow‐up. During this interval, 70% of participants had no hands‐on ERCP/EST experience, suggesting limited direct confounding by subsequent practice. However, routine gastrointestinal endoscopy may still have acted as a confounding factor.

## 5 Conclusion

A BESAT‐based structured educational program using a novel simulator improves simulator‐based EST performance and self‐confidence among gastroenterology trainees. The psychological gains achieved through this program were sustained over a 3‐month follow‐up period. By providing a safe environment for using actual devices and safely experiencing failures, this simulator‐based approach may serve as a useful complement to training in pancreatobiliary endoscopy.

## Author Contributions

Conception and design (**Yutaka Hatayama** and **Takeshi Kanno**); analysis and interpretation of the data. (**Yutaka Hatayama** and **Takeshi Kanno**); drafting of the article (**Yutaka Hatayama**, **Tetsuya Takikawa**, and **Takeshi Kanno**); critical revision of the article for important intellectual content (**Yutaro Arata**, **Ryotaro Matsumoto**, **Suguo Suzuki**, **Kaname Uno**, **Kazuhiro Kikuta**, and **Tomoyuki Koike**); final approval of the article (**Atsushi Masamune**).

## Conflicts of Interest

Dr. Takeshi Kanno has joint research funds from Denka Company and U‐A Company. However, these companies had no control over this work's interpretation, writing, or publication. The remaining authors declare no conflicts of interest.

## Funding

This work was supported by JSPS KAKENHI (Grant Nos. 24K23401, 25K13481, and 26K03047).

## Supporting information




**Supporting File 1**: deo270382‐supp‐0001‐SuppMat.docx

**Supporting File 2**: deo270382‐supp‐0001‐Video1.mp4

## Data Availability

Deidentified data and statistical code are available upon reasonable request from the corresponding author. **Study approval**: The study protocol was approved by the Ethics Committee of the Tohoku Medical Megabank Organization, Tohoku University, on March 15, 2021 (approval number 2020‐4‐195) and conducted in accordance with the Declaration of Helsinki. The trainees provided oral consent prior to the start of the study.
