# Supplementary material for: Bethesda Endoscopic Retrograde Cholangiopancreatography Skill Assessment Tool (BESAT)‐Based Simulator Training Using a Novel Dry Endoscopic Retrograde Cholangiopancreatography Model Improves Technical Proficiency and Self‐Efficacy: A Prospective Study
Source: DEN Open. 2026 Sep 1;7(1):e70382. doi: 10.1002/deo2.70382 (PMC13531230; doi:10.1002/deo2.70382)
Supplement: Supplementary file 1 [file DEO2-7-e70382-s001.docx]

**Supplementary Information 1. Detailed contents of the BESAT-based feedback**
The BESAT-based educational intervention focused on six technical points: (i) positioning alignment and maintenance to ensure a stable frontal view of the papilla; (ii) positioning and trajectory optimization for biliary cannulation; (iii) gentle manipulation to prevent trauma or deformity of the papillary orifice; (iv) proficient wire manipulation and maintenance of stable wire position; (v) sphincterotomy control and trajectory precision, focusing on the hooding fold and oral protrusion; and (vi) refinement of procedural judgment to optimize equipment and maneuver selection for safety and success.
